# Editorial: Aquatic Pharmacology, Volume II: Pharmacokinetics for Aquatic Species

**DOI:** 10.3389/fvets.2022.1039868

**Published:** 2022-10-17

**Authors:** Prapansak Srisapoome, Lisa A. Tell, Chi-Chung Chou

**Affiliations:** ^1^Department of Aquaculture, Faculty of Fisheries, Kasetsart University, Bangkok, Thailand; ^2^Department of Medicine and Epidemiology, School of Veterinary Medicine, University of California, Davis, Davis, CA, United States; ^3^Department of Veterinary Medicine, College of Veterinary Medicine, National Chung Hsing University, Taichung, Taiwan

**Keywords:** pharmacokinetics, antibiotics, aquatic, anesthetics, anti-fungal, NSAIDs

The second edition of Aquatic Pharmacology features six articles, of which five belong to the discipline of pharmacokinetics and one on the anti-fungal activity of disinfectants. Further breakdown of the topics in this edition, include three pharmacokinetic manuscripts on fluoroquinolones (Shan et al.; Song et al.; Yang et al.), one on an amphenicol antibiotic, florfenicol (Rairat et al.), and one on a non-steroidal anti-inflammatory drug (NSAID), meloxicam (Moron-Elorza et al.). Concerned species include crucian carp (*Carassius auratus gibelio*), yellow river carp (*Cyprinus carpio haematoperus*), Nile tilapia (*Oreochromis niloticus*), and large-spotted catsharks (*Scyliorhinus stellaris*). The lone non-pharmacokinetic submission was about the evaluation of the anti-oomycete activity of chlorhexidine gluconate against *Saprolegnia* spp. through molecular docking, *in silico* analysis, and determination of minimum inhibitory/fungicidal concentrations (Thakuria et al.). An interesting observation is that the second edition's content is exactly the same as in the first edition, where five of the six articles were related to pharmacokinetic research. Aside from possible influences from the editors' background, the collection of articles might again reflect the lack of clinical instruction on limited pharmaceutics available for aquatic species and the need to resolve these shortcomings. Therefore, it might be worthwhile to cast a deeper look into the unique features of pharmacokinetics in aquatic species, mainly fish, as they represent the main body of the two editions of aquatic pharmacology.

Pharmacokinetic studies can give rise to information critical for determining of dose, dosing interval, drug-drug interactions, and in food animals, the withdrawal times to assure efficient treatment and safeguard food from residual toxicity to humans. Such information should be tailored to matched animal species and drugs under specified conditions. This is especially true and even more critical for aquatic animals. Using farm fish as an example, spread-dosing with feed in their rearing environment renders higher dose variation. Drugs, either in parent or metabolized forms, stay in the environment where fish live, creating a continuous immersion effect and could further pollute their living environment and intoxicate surrounding non-target organisms. This creates a unique concern/feature for approval of medicines in aquatic species because risks associated with inappropriate uses, explicitly tissue residue violations, drug resistance, and environmental pollution, could be well above those in land animals. Furthermore, as fish are poikilothermic animals, their body temperature and metabolic rate fundamentally depend on water temperature. An increase in water temperature would result in increased metabolic rate, blood flow (and blood flow-dependent clearance), and drug-metabolizing enzyme activity; such that the pharmacokinetic behaviors of aquatic medications are largely dictated by rearing temperatures. This temperature-dependent pharmacokinetics warrants specification of the temperature in order to formulate a more accurate optimal dosage. To make matters worse, aquatic species are very diversified. Currently, the pharmacokinetic information in use is mainly derived from a few representative fish in the same “order” rather than direct study of specific species, which adds further imprecision to the pharmacokinetic aspects of clinical practice.

Consequently, approved medications for aquatic species are significantly falling behind terrestrial animals. Again, using antibacterials approved for fish as an example, approved numbers of antibiotics in most countries are below twenty; for instance, only 1 in Norway (1), 3 in the USA (2), 6 in China (3), 11 in Thailand (4), 12 in Japan (5), and 14 in Taiwan (6). The European Union has the most approvals of 29 antibiotics (7) due to diversified territorial backgrounds encompassing more than 25 union countries with their preferred regulations. Antibiotics approved by most countries include florfenicol, oxytetracycline, sulfonamides, oxolinic acid, and amoxicillin. This factual scenario highlights the hardship of the effective use of available drugs for infection control in fish.

In addition to antibacterials, published aquatic pharmacokinetic research also mainly concentrates on anti-infectives, including anthelmintics (8–10), antivirals (11, 12), and natural botanic products with anti-infective peroperties (13–15). Drugs relating to experimental or medical management of aquatic species such as NSAIDs (16–18) and anesthetics (19–21) are also topics for pharmacokinetic studies. The majority of fish species include those of economic importance, such as carp, Nile tilapia, catfish, rainbow trout, Atlantic salmon, gilthead seabream, European seabass, and grouper (22). Other non-fish species that are also covered include shrimp (23, 24), crab (25, 26), frog (27, 28), turtle (29, 30), and crocodile (31), which also to some extent carry economic implications. Although it doesn't have to go far to find some publications related to aquatic pharmacokinetics, the lack of knowledge in need is no doubt significant.

On the other hand, it is notable that other than traditional pharmacokinetic studies concerning drug bioavailability, tissue distribution, enzymes metabolism, withdrawal times, and factors affecting (ex. temperature and salinity) those processes, the population pharmacokinetics was also seen in this edition. Population pharmacokinetic studies evaluate drug disposition features in a population, using a limited number of samples per study subject and considering the influence of diverse clinical/pathophysiological factors and individual variability on pharmacokinetics. It can be a tool to optimize the determination of efficacy and safety of drugs. The application of this methodology allows the establishment of withdrawal intervals tailored to the clinical or production conditions of populations or individuals such that a safer food supply is more likely for a wide variety of dose and off-label clinical uses (32). This approach is a delightful welcome that could bolster the benefit of pharmacokinetic research at reduced cost and labor.

As indicated in our first editorial, out of more than 300 veterinary and aquatic science journals listed in the science citation index (SCI), no single journal is dedicated specifically to pharmacological research in the aquatic species, not to mention any specialization in pharmacokinetics. While such journal is a far reach even for land animals, we hope the completion of this special topic edition could provoke the idea for a future journal section dedicated to the collection of articles pertaining to aquatic pharmacokinetics in the Frontiers in Veterinary Science-Veterinary Pharmacology and Toxicology.

## Author contributions

All authors have made a substantial, direct, and intellectual contribution to the work and approved it for publication.

## Conflict of interest

The authors declare that the research was conducted in the absence of any commercial or financial relationships that could be construed as a potential conflict of interest.

## Publisher's note

All claims expressed in this article are solely those of the authors and do not necessarily represent those of their affiliated organizations, or those of the publisher, the editors and the reviewers. Any product that may be evaluated in this article, or claim that may be made by its manufacturer, is not guaranteed or endorsed by the publisher.
